# Leukocyte Image Segmentation Using Novel Saliency Detection Based on Positive Feedback of Visual Perception

**DOI:** 10.1155/2018/5098973

**Published:** 2018-02-01

**Authors:** Chen Pan, Wenlong Xu, Dan Shen, Yong Yang

**Affiliations:** ^1^China Jiliang University, Hangzhou, Zhejiang 310018, China; ^2^Department of Hematology at the First Hospital Affiliated to Medical College, Zhejiang University, Hangzhou, Zhejiang 310003, China; ^3^School of Information Technology, Jiangxi University of Finance and Economics, Nanchang 330013, China

## Abstract

This paper presents a novel method for salient object detection in nature image by simulating microsaccades in fixational eye movements. Due to a nucleated cell usually stained that is salient obviously, the proposed method is suitable to segment nucleated cell. Firstly, the existing fixation prediction method is utilized to produce an initial fixation area. Followed EPELM (ensemble of polyharmonic extreme learning machine) is trained on-line by the pixels sampling from the fixation and nonfixation area. Then the model of EPELM could be used to classify image pixels to form new binary fixation area. Depending upon the updated fixation area, the procedure of “pixel sampling-learning-classification” could be performed iteratively. If the previous binary fixation area and the latter one were similar enough in iteration, it indicates that the perception is saturated and the loop should be terminated. The binary output in iteration could be regarded as a kind of visual stimulation. So the multiple outputs of visual stimuli can be accumulated to form a new saliency map. Experiments on three image databases show the validity of our method. It can segment nucleated cells successfully in different imaging conditions.

## 1. Introduction

Microscopic leukocyte analysis is a powerful diagnostic tool for many types of diseases for which it is vital to recognize and count different lineages and maturity levels of leukocytes. Computer-aided automatic analysis not only saves manpower and time but also reduces human error. The most important step in automatic image analysis is segmentation. Human leukocytes (WBCs) are colorless. Blood and bone marrow smears are conventionally prepared with Wright-Giemsa stain in order to visualize obviously and identify WBCs. However, different smear preparation and imaging conditions may result in large biases and changes in image color. It is difficult to segment entire leukocyte populations since color distributions may be uncertain.

Nature image is typical unstructured data. Modeling such data via machine learning has been a hotspot for decades. In recent years, two classes' learning-based algorithms, bottom-up and top-down, which composed with shallow and deep neural networks, respectively, are widely used to solve the segmentation problem. In bottom-up framework, literatures [1, 2] had proposed data-driven methods to segment leukocyte image via “pixel sampling-learning-classification” procedure based on shallow network (SVM or ELM). However, those algorithms have some priori restrictions. For example, the algorithm assumes that the nuclei of WBCs are surrounded by cytoplasm and are always deeply stained so that the object intensity is low while the background intensity is bright. Moreover, preparing the training samples is very critical in those methods. Because if the samples are not good (or not pure) enough, the learning-based algorithm may output undesired object. It is a challenge to solve such noise-sample-sensitive problem in a learning-based framework. The state-of-the-art top-down model is developed from deep learning, which has been successfully used for image segmentation [3]. Deep learning-based algorithm reflects the best performance in many applications so far, since that can deal with object-level or image-level representative features from training samples. However, deep networks often have huge parameters than the shallow one, so that they need massive labeled sample data to tune parameters repeatedly in training. The most of the existing top-down learning-based methods are time-consuming in off-line training process or labeling positive samples manually.

Due to nucleated cell usually stained with salient color, Zheng et al. [4] firstly locate nuclei by saliency detection method and then extract the nucleated cell by maker-controlled watershed. However, saliency detection in Zheng's method was too simple and may be out of date. It is necessary to find some new ways to update them.

In general, without prior knowledge and effective samples, many segmentation methods may fail in practice. In addition, we note that the information is often feedforward and lacks feedback process in most bottom-up or top-down models. It greatly differs from human vision. That may be one of the reasons the performance of machine vision is far from that of human vision.

Human accepts attention by making a series of eye movements. There are two forms of eye movement: saccades and microsaccades. (1) In saccade stage, human eyes aim to find candidate object so it makes sharply shifts in the whole field of view. (2) While candidates are identified as target, the eyes will make a series of dense tiny movements that is called microsaccades around the target for the purpose of intensify objects and inhibit noises. Continuous microsaccades will lead to visual fading [5], and the eye movement will switch to the stage of saccades to find new objects. The integration of saccades and microsaccades contributes to the quick and efficient performance of human vision system.

Motivated by the above reasons, this paper presents a novel saliency detection framework by simulating microsaccades and visual fading, without prior knowledge and labeled samples. We construct a positive feedback loop to focus on fixation area and intensify objects repeatedly. Ensemble of polyharmonic extreme learning machine (EPELM) [6, 7] is utilized to simulate the human neural system to produce visual stimulus. Depending on sampling from previous fixation area (input), training EPELM model using the samples, and classifying image pixels by EPELM, new fixation area can be output in iteration. If the input and output fixation area were similar enough, it indicates that the perception is saturated and the iteration should be terminated. The final fixation area is the segmentation result in our method. Experimental results show that new saliency method with positive feedback loop can achieve better performance and can greatly improve the performance of the existing saliency detection methods.

In summary, the main contributions of our work are as follows. (1) We propose a novel learning-based algorithm to detect salient objects depending on bottom-up saliency. It is good to segment stained leukocyte without any prior knowledge and labeled samples. (2) A positive feedback module based on EPELM is presented which focuses on fixation area for the purpose of intensifying objects, inhibiting noises, and promoting saturation in perception. Positive feedback of perception may be indispensable in saliency detection.

In the rest of the paper, we introduce the works of saliency detection that are heavily related to our approach in Section 2. Then we describe our algorithm in Section 3 and finally discuss the experimental result in Section 4.  Section 5 is the conclusions.

## 2. Related Works

Visual attention is a remarkable capability of early primate visual system, which helps human complete scene analysis in real-time with limited resources. Inspired by it, various computational models, which are called saliency models, have been proposed according to the psychological and neurobiological findings. Saliency model aims to identify the most salient foreground object from the background, and this problem in its essence is a figure/ground segmentation problem. In general, saliency detection can also be grouped into two categories: top-down methods and bottom-up methods. Bottom-up methods are rapid, data-driven, and task-independent, which construct saliency maps based on low-level visual information, such as pixel level or super pixel level. Due to the absence of high-level knowledge, all bottom-up methods rely on assumptions about the properties of objects and backgrounds. The widely utilized assumptions could be contrast prior, boundary prior, center prior, background prior, and so on. In contrast, top-down approaches are slower, volition-controlled, and task-driven and require supervised learning based on training samples with manual labels.

The classic bottom-up computational model is Borji et al.'s method [8], which gets saliency values of each pixel by center-surround contrast. Hou and Zhang [9] use the residual Fourier amplitude spectrum to form saliency map. Both of the above two models aim to predict human fixation points; hence, saliency maps computed by these models are spatially discontinuous. While at the same time, models for the purpose of salient region detection have been proposed in [10], Goferman et al. proposed a saliency model based on context-aware, and Cheng et al. [11] presented global contrast-based saliency computation methods, called histogram-based contrast (HC) and spatial information-enhanced region-based contrast (RC). These types of model can generate saliency maps with fine details and high resolution. Literature [8] indicates that models for salient region detection shown actual advantage in contrast with models for fixation prediction in terms of various computer vision applications.

Recently, many learning models are proposed for saliency computation. Methods based on supervised learning have emerged [12], and these approaches use large fine annotation images to train saliency model, which is a typically knowledge-driven approach. At the same time, there are some unsupervised learning approaches [13]. All of the above methods either rely on large manual-labeled dataset and off-line training or lie on numbers of parameters set for modeling.

In the bottom-up framework, [14–16] presented some effective ways to to train a set of weak classifiers based on initial saliency maps and then obtained a strong model by integrating the weak classifiers or their results. In their approaches, multiple types of classifiers, multiscale analysis, and graph cut algorithm could be taken together, such as Na et al. [14] presented BL algorithm which detect objects via bootstrap learning of SVMs. Huang et al. [15] presented MIL algorithm depending on object proposals and multiple instance learning using SVMs. And in [16], Zhang et al. presented salient object detection based on ELM. Those methods are very attractive to us since the learning-based approach is similar to human brain, and their results are very close to human perception. However, we noticed that those methods are almost no reference to human visual mechanism. They implement function from computing rather than simulating human vision. It is time-consuming because of multiscale analysis and multimodel parallel computing that may lose the speed of bottom-up model. In addition, the information is also feedforward and lacks feedback process in those methods. There may be large room to improve them.

## 3. Method

Our method consists of four main components:

- Generating a gaze area
- Forming a preliminary object using coarse saliency map by learning
- Suppressing the background
- Intensifying object to form a sense of saturation by learning-based positive feedback

**Figure 1:**
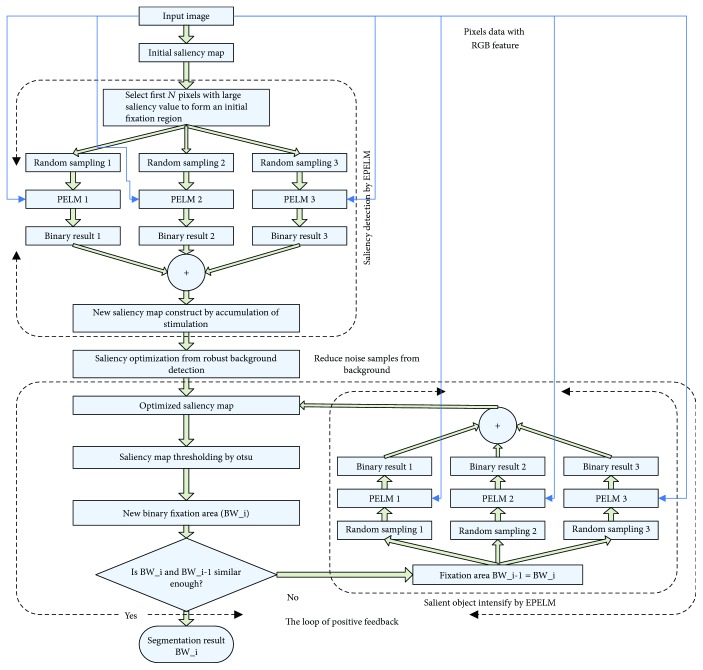
The framework of the proposed method.

The framework of our method is illustrated in Figure 1.

**Figure 2:**
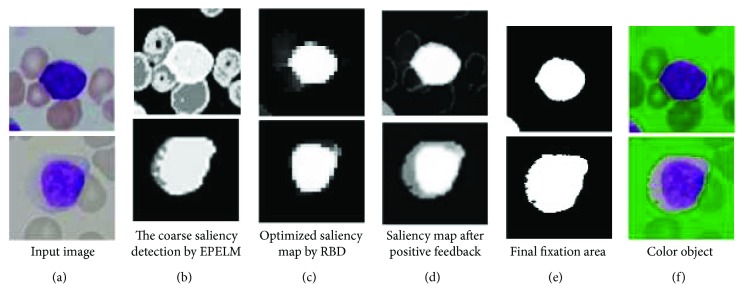
The main pipeline of our method.

The key steps are listed as follows. See Figure 2, the main pipeline of our method.

Step 1. An initial saliency map is made from input image by SR algorithm.
Step 2. Use coarse saliency detection by EPELM learning:
(1) Sort pixels according to the saliency, and select the first n pixels with large value (*n* = 100 in our experiment).
(2) The selected pixels form a minimum rectangle box containing them. Inside the box is the fixation area, so the outside is the nonfixation area.
(3) Random sample *m* pixels with high gradient are from the fixation area (positive samples). And random sample equal pixels are from the nonfixation area (negative samples) (*m* = 500 in our experiment).
(4) Use training EPELM using the positive and negative pixels with RGB features.
(5) Classify image pixels by EPELM. Each binary output of PELM is regarded as single stimulation, could be normalized, and is added to form a coarse saliency map.
Step 3. RBD algorithm is used to reduce the noise in the coarse saliency map, by background detection and saliency optimization.
Step 4. Intensify objects using positive feedback loop:
(1) Threshold the optimized saliency map to make new binary fixation area (BW_i).
(2) If BW_i-1 has been existed, then judge whether BW_i is similar enough to BW_i-1. If true, go to step 5 (break the loop); else, do the next step.
(3) Use Saliency detection by EPELM learning (same as step 2). Each binary output of PELM could be normalized and added to the saliency map.
(4) Return to step 1 in the current step.
Step 5. The final segmentation result is BW_i (end).

### 3.1. The Function of SR and RBD Algorithms

SR (spectrum residual) method was presented by Hou and Zhang [9], which aims to predict human fixations and often produces blob-like and sparse saliency map corresponding to the human fixation spots on scenes. Let *I*(*x*) be the image, *x* be the pixel position, *F*() be the Fourier transformation; then

(1) $$\begin{array}{c} A\left(f\right)=\left|F\left(\left[I\left(x\right)\right]\right)\right|,\\ P\left(f\right)=\varphi \left(F\left[I\left(x\right)\right]\right),\\ L\left(f\right)=\log\left(A\left(f\right)\right),\\ R\left(f\right)=L\left(f\right)-h_{n}\left(f\right)*L\left(f\right),\\ SR\left(x\right)={\left|F^{-1}\left[\exp\left\{R\left(f\right)+jP\left(f\right)\right\}\right]\right|}^{2}, \end{array}$$

where *A*(*f*) is the amplitude spectrum of image, *P*(*f*) is the phase spectrum of image, *L*(*f*) is the log of amplitude spectrum, *R*(*f*) represents residual Fourier amplitude spectrum, SR(*x*) is the saliency map, *φ*() is the operation to extract phase, and *h*_*n*_(*f*) is an average operator.

The salient points detected by SR often have strong correlation with eye gaze spots. Besides, SR is very similar to human perception since saliency map may change when the scale of the image changes. And it is one of the fastest fixation prediction algorithms [8]. So we select it to simulate human fixation.

**Figure 3:**
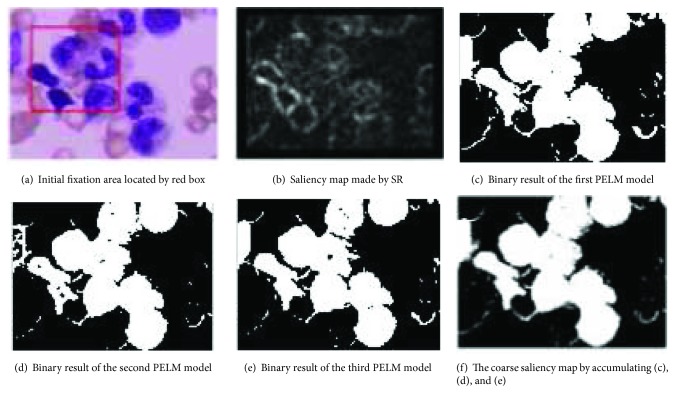
(a-b) Initial fixation area made by SR; (c–f) the coarse saliency map made by PELM learning.

In our method, we firstly provide an initial fixation area using SR, then sampling from there, and learning by EPELM. Multiple random sampling may be equivalent to the micro scan in the fixation region. Because the training samples are few (*m* = 500 in this paper), the EPELM classifier can be trained in real-time. After that, those models are used to classify image pixels into classes of object or background. The binary output of every PELM model could be treated as a kind of stimulus just like neuron firing in human brain. Multiple outputs of PELMs could be accumulated together and normalized to form a new coarse saliency map. Figures 3(a)–3(f) show an example.

Since the initial fixation area is often rough (see the red box in Figure 3(a)), so that there is a lot of noise in the positive and negative samples. Such noise samples may easily lead to undesired output. Although we accumulate the learning-based results, it is not enough to decrease the bad influence of background pixels to foreground. In order to erase the error caused by noise samples, the coarse saliency map needs to be optimized further by suppressing the background.

RBD (saliency optimization from robust background detection) was proposed by Zhu et al. [17], which belongs to salient object detection models and attempts to highlight the whole salient object by suppressing the background. Zhu et al. proposed a robust background measure, called boundary connectivity. It characterizes the spatial layout of image regions with respect to image boundaries. It is defined as

(2) $$\begin{array}{c} BndCon=\frac{\left|\left\{p\;|\;p\in R,p\in Bnd\right\}\right|}{\sqrt{\left|\left\{p\;|\;p\in R\right\}\right|}}, \end{array}$$

where *p* is an image patch and Bnd is the set of image boundary patches. It has an intuitive geometrical interpretation: it is the ratio of a region's perimeter on the boundary to the region's overall perimeter or square root of its area.

Zhu et al. presented an approach depending on superpixels to compute background probability by boundary connectivity.

(3) $$\begin{array}{c} \omega_{i}^{bg}=1-\exp\left(\frac{{BndCon}^{2}\left(p_{i}\right)}{2\sigma_{BndCon}^{2}}\right). \end{array}$$

The salient object detection problem in their model is regarded as the optimization of the saliency values of all image superpixels. An optimization framework to integrate an initial saliency map with the background measure is presented. The objective cost function is designed to assign the object region value 1 and the background region value 0, respectively. The optimal saliency map is then obtained by minimizing the cost function.

Let the saliency values of *N* superpixels be {*s*_*i*_}_*i*=1_^*N*^, and the cost function is

(4) $$\begin{array}{c} cost=\overset{N}{\underset{i=1}{\sum }}\omega_{i}^{bg}s_{i}^{2}+\overset{N}{\underset{i=1}{\sum }}\omega_{i}^{fg}{\left(s_{i}-1\right)}^{2}+\sum_{ij}\omega_{ij}{\left(s_{i}-s_{j}\right)}^{2}. \end{array}$$

There are three terms which define costs from different constraints. *ω*_*i*_^bg^ is the background probability, *ω*_*i*_^fg^ is the foreground probability often represented by initial saliency map, and *ω*_*ij*_ is the smoothness term which encourages continuous saliency values which is used to erase small noise in both background and foreground terms. We select optimal value of {*s*_*i*_}_*i*=1_^*N*^ to minimize the cost.

**Figure 4:**
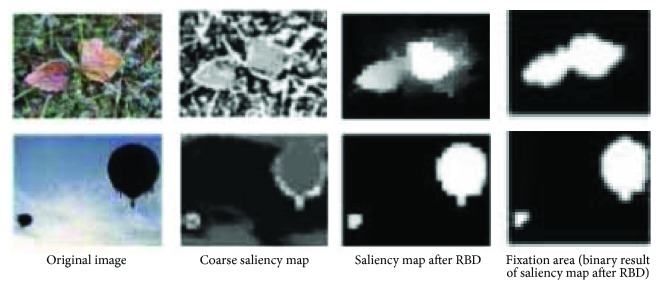
The function of RBD algorithm: suppressing the background pixels.

In our method, *ω*_*i*_^fg^ is the coarse saliency map produced by our method from step 1 to step 2. It could be carried into RBD algorithm to eliminate noise effects of background. See Figure 4. Pure samples will yield more precision model and binary result through the followed positive feedback.

### 3.2. Training of EPELM

ELM (extreme learning machine) has been widely used as a fast learning method for feedforward networks with a single-hidden layer [6]. Recently, Zhao et al. [7] extended it for more stable performance, which is called EPELM (ensemble of polyharmonic extreme learning machine). It has shown good performance in human face recognition. Due to polyharmonic mechanism, EPELM is an effective way to deal both kinds of scattered date with rapid changed and slow variations. Different from traditional learning algorithms which based on the gradient descent techniques for parameter optimization, EPELM sets its inner weights randomly and needs no iterative training. It can be trained on-line with small sample sets and needs not tuned with any parameter. So we use EPELM for learning-based saliency detection.

For a given set of training samples {(*x*_*i*_, *t*_*i*_)}_*i*=1_^*N*^ ⊂ *R*_*n*_ × *R*_*m*_, the output of a PELM with *L* hidden nodes can be written by

(5) $$\begin{array}{c} f^{r}\left(x\right)=\overset{L}{\underset{i=1}{\sum }}{\beta^{r}}_{i}\cdot G\left({a_{i}}^{\left(r\right)},{b_{i}}^{\left(r\right)},x\right)+P\left(x\right),x\in R^{n}, \end{array}$$

where *a*_*i*_ and *b*_*i*_ are the inner weights of input node to hidden node. *β*_*i*_ is the output weights of hidden node to output node. The inner weights in this model are randomly assigned. *G*(*a*_*i*_, *b*_*i*_, *x*) is the output of *i*th hidden note. *p*(*x*) is a polynomial with low degree, which can deal with the type of data with slow variations. Output weights *β* can be computed by the following formula:

(6) $$\begin{array}{c} \overset{\wedge }{\beta^{r}}=H^{+}T, \end{array}$$

where *H* ^+^ is the Moore-Penrose pseudoinverse of the hidden layer output matrix, and *T* = [*t*_1_, *t*_2_,…,*t*_*n*_]^*T*^.

For the aim of gaining more stable model, we integrate numbers of PELM. The parameter *p* denotes the number of PELM grouped in the EPELM. The function for EPELM is (*p* = 3 in our experiment)

(7) $$\begin{array}{c} f\left(x\right)=\frac{1}{p}\overset{p}{\underset{r=1}{\sum }}f^{r}\left(x\right). \end{array}$$

**Figure 5:**
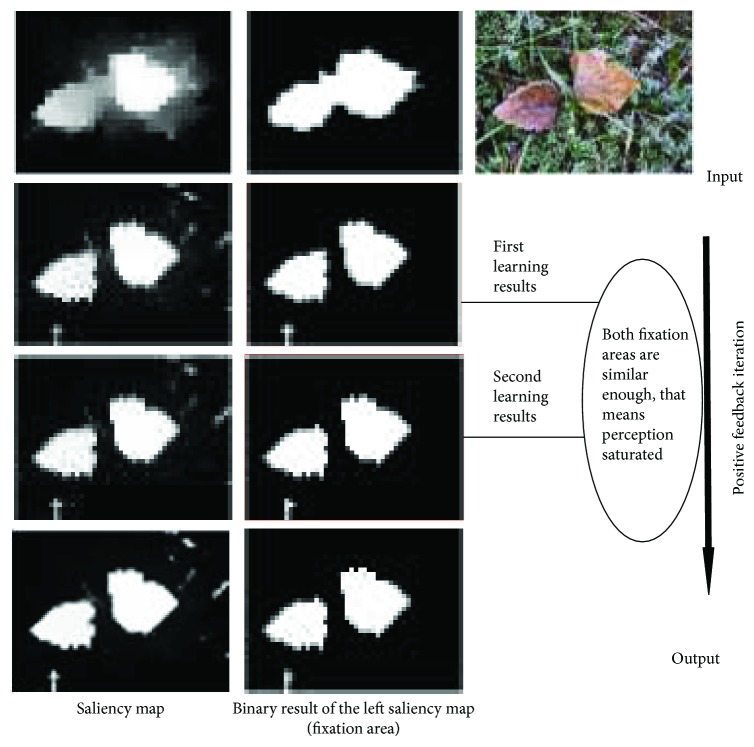
The positive feedback accelerates the process for the perception to become saturated.

In this paper, EPELM can be treated as neural system of human brain to accept stimulus and output new one. The function of positive feedback loop based on EPELM is illustrated in Figure 5. It is easy and quick that visual perception becomes saturated in positive feedback loop.

## 4. Experimental Results and Analysis

### 4.1. Dataset

To evaluate the performance of our algorithm, we have chosen three widely used datasets. SED2 contains 100 nature images with two salient objects. Every image in the dataset was finely labeled manually for the purpose of saliency detection and image segmentation. ALL-IDB1 and ALL-IDB2 are the acute lymphoblastic leukemia image database [18].

### 4.2. Implementation Details

In this paper, input image with large size should be downsampled to 64^∗^64 for fixation prediction and salient object detection, because SR algorithm is sensitive to image size, and 64 pixels of input image may be a good estimation of majority images. More importantly, reducing the size of image can save running time sharply. The number of superpixels of RBD could be set to 100 or 150. It is not sensitive to our method.

The number of positive and negative samples is set to 500 in sampling. And the number of hidden nodes of PELM may be linked to the dimension of pixels feature and could be set to 5~30 in this paper. In order to control the loop of positive feedback, *F*-measure is used to measure the similarity between *BW_i* and *BW_i-1*. And *F* = 0.95 means both areas are similar enough in our experiment.

### 4.3. Evaluation Measures

We perform both quantitative and qualitative evaluations for our approach. For quantitative evaluation, we use recall, precision, and *F*-measure. *F*-measure jointly considers recall and precision. For a saliency map *S*, we first covert it to a binary mask *M* by thresholding using a fixed threshold which changes from 0 to 255. On each threshold, a pair of P/R scores is computed to form PR-curve and to describe the performance of model at different situations. Recall and precision can be computed by the following function.

(8) $$\begin{array}{c} P=\frac{\left|M\cap G\right|}{\left|M\right|},\\ R=\frac{\left|M\cap G\right|}{\left|G\right|}, \end{array}$$

where *G* denotes the ground truth, and *F*-measure can be defined as follows:

(9) $$\begin{array}{c} F=\frac{\left(1+\beta^{2}\right)*P*R}{\beta^{2}*P+R}. \end{array}$$

As suggested by the literature [8], *β* ^2^ is set to 0.3 to enhance the effect of precision. The more the *F* value is, the better the performance. We take the average *F*-measure of each database as the final *F*-measure.

### 4.4. Experimental Results

#### 4.4.1. Nature Image Saliency Detection and Segmentation

**Figure 6:**
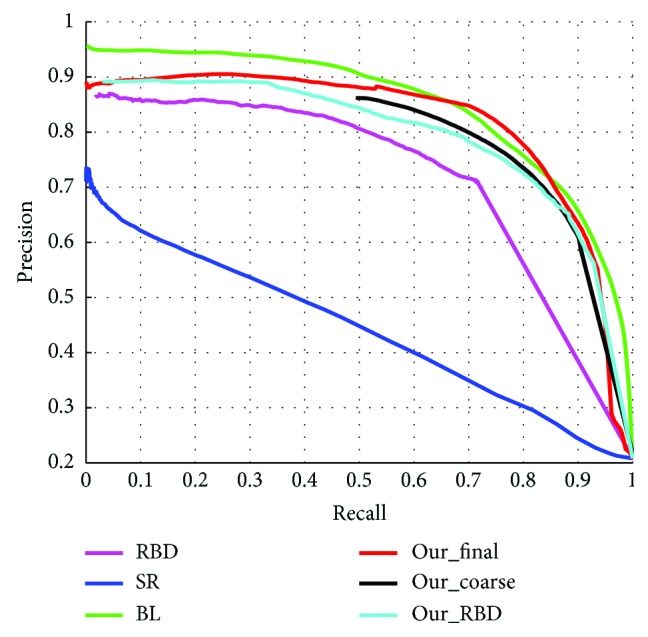
PR curves of ours and four compared algorithms in SED2.

We firstly test our approach in SED2 database. Three models were compared which are state-of-the-art or closely related to our approach: BL [14], SR [9], and RBD [15]. PR curves of compared methods are shown in Figure 6. In PR curves, Our_final means saliency map output from the positive feedback loop; Our_coarse is the coarse saliency map output by first learning; Our_RBD is optimized saliency map after RBD. Other saliency maps are represented by algorithm names.

Figure 6 shows that the top-left of the BL's curve is higher than the others. It means that BL's saliency map is more detailed and smooth. However, Our_final is more than BL in the middle of the PR curve that illustrates the ability of our method to grasp the whole object is better than BL. Besides, Our_coarse and Our_RBD are higher than the original RBD and SR. Although the curves of Our_coarse and Our_RBD are little lower than those of the BL, Our_final achieves good result after the positive feedback. Obviously, positive feedback has played a decisive role in improving performance.

Table 1 shows average *F*-measures for the 4 methods. Those results show that our method has the best performance, followed by BL and RBD, and SR is worst. It is also shown that the performance of SR and RBD can be improved effectively by adding learning-based positive feedback.

**Figure 7:**
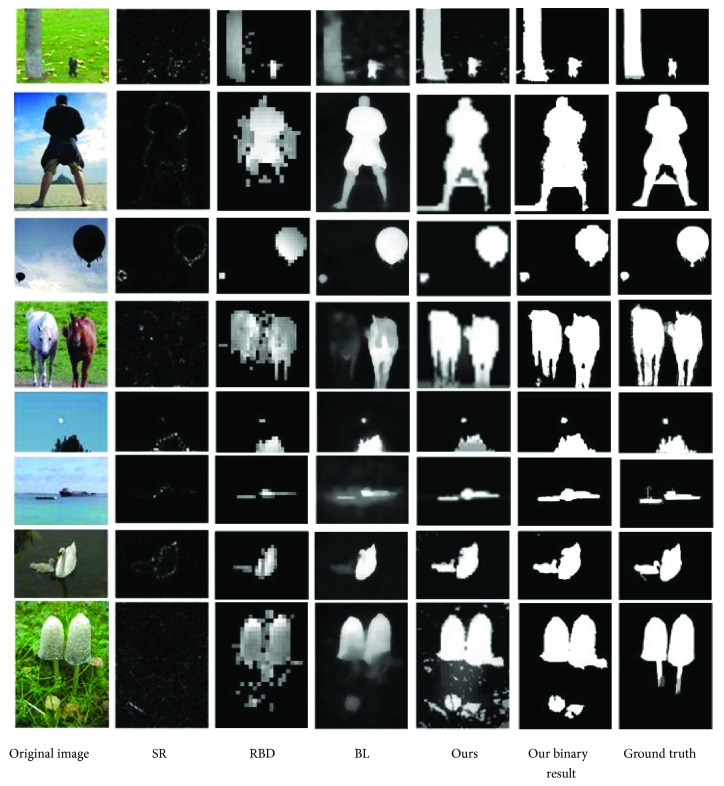
Comparison of partial experimental results in SED2.

Figure 7 shows part of the images in the SED2 and their saliency maps obtained by the 4 methods. These results show that the BL saliency map is better in smoothness and detail, and our method is better in overall perception. It should be noted that SR, RBD, and our methods reduce the size of original image in saliency detection and their saliency maps are rougher than BL's one. From the view of qualitative evaluation, it is clear that the binary object mask detected by our method is closer to the ground truth.

#### 4.4.2. Leukemia Image Segmentation

ALL-IDB1 contains 108 images with large field of vision, each image includes many WBCs. Some of them may overlap and touch together. ALL-IDB2 contains 260 images with small field of vision, and each of them only contains a nucleated cell. The difficulty lies in that conventional methods are hard to extract the entire leukocyte populations, due to the color of cytoplasm of WBCs often close to that of the background.

Two methods were compared with our approach: marker-controlled watershed and Reference [2]'s method. The former performs flooding operation according to the selected markers and the gradient. The latter firstly finds the deep stained nucleus of WBCs by thresholding and then does sampling around the fixation area and learning/classification by SVM/ELM. We sketched the outline of the nucleated cells in the image as ground truth. The average *F*-measures are shown in Table 2.

Our method gets the highest score in ALL-IDB2, while slightly worse than Reference [2]'s method in ALL-IDB1. Watershed-based method is worst in both datasets.

**Figure 8:**
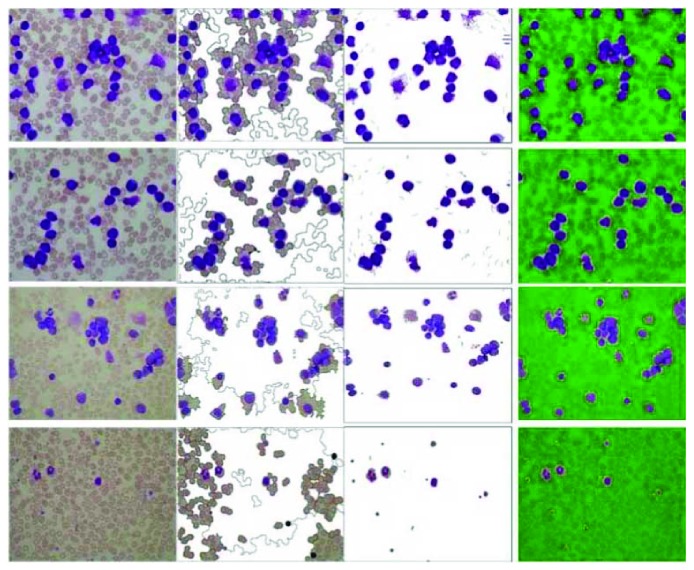
Comparison of partial experimental results in ALL-IDB1.

**Figure 9:**
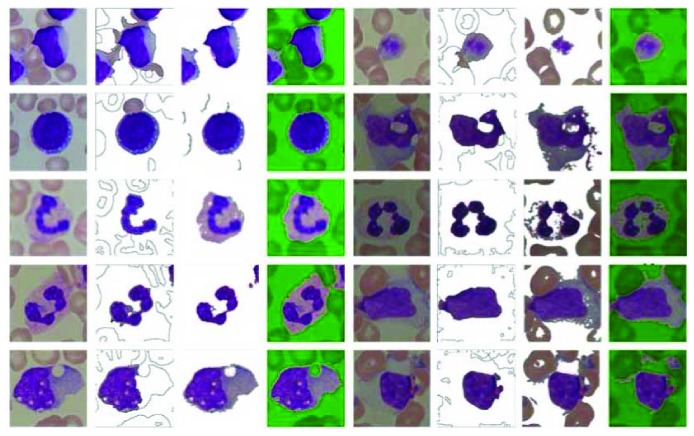
Comparison of partial poor results in ALL-IDB2.

Partial experimental results are listed in Figures 8 and 9. As can be seen from these examples, our method is successful in ALL-IDB2. In which only one stained cell exists, so the detail of the object may be well preserved in our method. In ALL-IDB1, a large number of stained cells gathered together may limit the performance of our method. While Pan's method without positive feedback procedure may be more appropriate to deal with this situation.

In ALL-IDB2, only one segmentation result is not ideal in our method. The segment result of this image losts most cytoplasm (shown in Figure 9, the right side of the last row); however, even the human eyes are prone to error in this image. These examples show that our approach is much close to human perception.

### 4.5. Discussion

The method of “pixel sampling-learning-classification” was proposed previously in [2]. It works well in good control condition. It needs be noted that the framework of the above method is very similar with step 2 in our method, in which shallow networks are parallelly arranged without any feedback. It is a noise sample-sensitive method if the training samples are not well prepared according to the prior knowledge. Our method could be regarded as an improved version developed from the technique of [2]. We presented an effective way to deal with the noise sample-sensitive problem by background-suppressing and learning-based positive feedbacks.

Our method also differs from Na's works especially in simulating human vision. Na's team tries to train a set of weak classifiers based on initial saliency maps and then obtains a strong model by integrating the weak classifiers. The final output relies on the strong model. They take boosting and parallel strategy to group weak classifiers, but without any feedback in their framework. In contrast, our method only focuses on the fixation region to accelerate the process for the object perception to become saturated, no matter how the classifier is weak or strong. In our method, the saliency map could be produced by accumulating the binary result in iteration and object could be output by thresholding the saliency map. By the way, multiscale analysis is not involved in our method. We just downsample image to a small size (64^∗^64) that can sharply speed the algorithm, while it does not decrease the performance.

## 5. Conclusions

This paper proposes a novel saliency region detection method based on machine learning and positive feedback of perception. Motivated by human visual system, we construct a framework using EPELM to process visual information from coarse to fine, to form a saliency map and extract salient objects. Our algorithm is data-driven totally and needs no any prior knowledge compared with the existing algorithms. Experiments on several standard image databases show that our method not only improves the performance of the conventional saliency detection algorithms but also segments nucleated cells successfully in different imaging conditions.

## Figures and Tables

**Table 1:** Average *F*-measure of four compared approaches in SED2.

Method	SR	RBD	BL	Ours
*F*-measure	0.700	0.8250	0.8342	0.8561

**Table 2:** Average *F*-measure of different approaches in ALL-IDB1 and ALL-IDB2.

	ALL-IDB1			ALL-IDB2		
Method	Watershed	Reference [2]'s method	Ours	Watershed	Reference [2]'s method	Ours
*F*-measure	0.60	0.89	0.86	0.56	0.82	0.95

## References

[1] Yu Z., Wong H. S., Wen G. (2011). A modified support vector machine and its application to image segmentation. *Image and Vision Computing*.

[2] Pan C., Park D. S., Yang Y., Yoo H. M. (2012). Leukocyte image segmentation by visual attention and extreme learning machine. *Neural Computing and Applications*.

[3] Long J., Shelhamer E., Darrell T. Fully convolutional networks for semantic segmentation.

[4] Zheng X., Wang Y., Wang G., Chen Z. (2014). A novel algorithm based on visual saliency attention for localization and segmentation in rapidly-stained leukocyte images. *Micron*.

[5] Matrin R. (2009). Microsaccades: small steps on a long way. *Vision Research*.

[6] Huang G.-B., Wang D. H., Lan Y. (2011). Extreme learning machines: a survey. *International Journal of Machine Learning and Cybernetics*.

[7] Zhao J. W., Zhou Z. H., Cao F. L. (2014). Human face recognition based on ensemble of polyharmonic extreme learning machine. *Neural Computing and Applications*.

[8] Borji A., Cheng M. M., Jiang H. Z., Li J. (2015). Salient object detection: a benchmark. *IEEE Transactions on Image Processing*.

[9] Hou X. D., Zhang L. Q. Saliency detection:a spectral residual approach.

[10] Goferman S., Manor L. Z., Tal A. (2012). Context-aware saliency detection. *IEEE Transactions on Pattern Analysis and Machine Intelligence*.

[11] Cheng M. M., Mitra N. J., Huang X., Torr P. H., Hu S. M. (2015). Global contrast based salient region detection. *IEEE Transactions on Pattern Analysis and Machine Intelligence*.

[12] Shi J., Yan Q., Xu L., Jia J. (2016). Hierarchical image saliency detection on extended CSSD. *IEEE Transactions on Pattern Analysis and Machine Intelligence*.

[13] Siva P., Russell C., Xiang T., Agapito L. Looking beyond the image: unsupervised learning for object saliency and detection.

[14] Na T., Huchuan L. U., Xiang R., Yang M.-H. Salient object detection via bootstrap learning.

[15] Huang F., Qi J., Lu H., Zhang L., Ruan X. (2017). Salient object detection via multiple instance learning. *IEEE Transactions on Image Processing*.

[16] Zhang L., Li J., Lu H. (2016). Saliency detection via extreme learning machine. *Neurocomputing*.

[17] Zhu W., Liang S., Wei Y., Sun J. Saliency optimization from robust background detection.

[18] Labati R. D., Piuri V., Scotti F. ALL-IDB: the acute lymphoblastic leukemia image database for image processing.

